# Mechanistic Insights Into Photobiomodulation for Primary Dysmenorrhea: A Narrative Review

**DOI:** 10.7759/cureus.104721

**Published:** 2026-03-05

**Authors:** Elizabeth Pelevin, Lauren Pabian, Sloane Auerbach, Amanda Ballard, Jessica Goempel, Lige Jiang, Justin Oro, Jacob Searles, Kaley Sloan, Emily Walter, Andrew Bouras, Robin J Jacobs

**Affiliations:** 1 Medicine, Nova Southeastern University Dr. Kiran C. Patel College of Osteopathic Medicine, Fort Lauderdale, USA

**Keywords:** cytochrome c oxidase, low-level light therapy (lllt), mitochondrial function, non-pharmacological therapy, pain management, photobiomodulation, primary dysmenorrhea, prostaglandins

## Abstract

Primary dysmenorrhea is widely recognized as a prevalent source of menstrual pain among individuals of reproductive age, with some patients reporting limited symptom relief with commonly used pharmacologic treatments. Photobiomodulation (PBM), including low-level light therapy in the red-light spectrum (approximately 610-630 nm), has emerged as a nonpharmacologic modality that has been explored in clinical research settings for dysmenorrhea. However, the biological mechanisms potentially underlying PBM-associated symptom improvement in this context are not fully elucidated, which may limit optimization of treatment parameters and broader clinical application. This narrative review synthesizes existing literature describing proposed molecular and cellular mechanisms through which PBM may influence dysmenorrhea-related pathways, with particular attention to inflammatory mediators, lipid metabolism, and implications for wavelength-specific treatment design. A narrative synthesis approach was employed, integrating findings from clinical studies that included biomarker assessments with established experimental and theoretical literature on PBM mechanisms. Sources reviewed included controlled clinical studies reporting biochemical outcomes, metabolomic investigations, and foundational mechanistic research. No new human or animal data were generated for this review. Prior experimental and translational work suggests that red-light PBM may interact with mitochondrial components involved in cellular energy processes, including cytochrome c oxidase, with downstream effects on cellular signaling pathways. In clinical and laboratory contexts, PBM exposure has been associated with changes in inflammation-related biomarkers, including prostaglandin-associated measures. Metabolomic studies further describe alterations in lipid-related metabolic pathways implicated in inflammatory processes, such as glycerophospholipid, linoleic acid, and arachidonic acid metabolism, with some findings suggesting potential wavelength-dependent effects. Collectively, these observations are compatible with the current understanding of dysmenorrhea pathophysiology and provide a plausible biological framework for reported clinical observations. In summary, PBM may influence dysmenorrhea-related symptoms through interconnected effects on mitochondrial signaling, inflammatory mediator activity, and lipid metabolism. Treatment protocols utilizing red-light wavelengths applied to lower abdominal or sacral regions are biologically plausible based on known optical and tissue interaction principles. Future research may benefit from systematic evaluation of dose parameters, standardized biomarker outcomes, and mechanistically informed approaches to patient selection.

## Introduction and background

Prostaglandin-mediated pathophysiology of primary dysmenorrhea

Primary dysmenorrhea (PD) is defined as recurrent menstrual pain occurring in the absence of identifiable pelvic pathology, affecting an estimated 45-95% of reproductive-age women worldwide [1,2]. The condition represents a leading cause of work and school absenteeism, with up to 29% of affected women experiencing severe, debilitating pain [1]. The fundamental pathophysiology centers on excessive prostanoid production via the cyclooxygenase (COX) pathway. As progesterone levels decline at the end of the luteal phase, phospholipase A2 is released from degrading lysosomes, initiating arachidonic acid metabolism and subsequent prostaglandin synthesis [2].

Prostaglandin F2α (PGF2α) and prostaglandin E2 (PGE2) are the primary mediators of dysmenorrheal pain. PGF2α induces intense uterine smooth muscle contractions and vasoconstriction, while PGE2 sensitizes afferent nerve endings to pain-producing stimuli [2,3]. The resulting uterine ischemia triggers anaerobic metabolism, lactate accumulation, and C-fiber activation, producing the characteristic cramping pain. Critically, endometrial prostaglandin concentrations correlate directly with dysmenorrhea severity, establishing prostaglandin overproduction as both cause and therapeutic target [1,4].

Treatment gaps and the need for mechanistically informed alternatives

First-line pharmacological management relies on COX-1/COX-2 inhibitors, which are nonsteroidal anti-inflammatory drugs (NSAIDs), to suppress prostaglandin synthesis, with combined oral contraceptives (COCs) as the primary alternative [5,6]. However, these approaches face significant limitations. Approximately 18% of dysmenorrhea patients demonstrate NSAID resistance, defined as failure to achieve adequate pain relief despite appropriate dosing and timing [7]. NSAIDs also carry risks of gastrointestinal adverse effects and are contraindicated in patients with renal impairment or aspirin-sensitive asthma [5]. COCs, while effective, are contraindicated in women with migraine with aura, thromboembolic risk factors, or those desiring pregnancy, and are not preferred by all patients due to hormonal concerns [6].

Non-pharmacological alternatives, including heat therapy, exercise, and transcutaneous electrical nerve stimulation (TENS), have demonstrated modest efficacy but lack robust evidence bases [8]. This therapeutic landscape, characterized by treatment resistance, contraindications, and patient preferences for non-pharmacological options, creates a clear need for mechanistically understood alternatives that can be rationally optimized.

Photobiomodulation: established mechanisms and emerging applications

Photobiomodulation (PBM) involves the application of red (600-700 nm) or near-infrared (700-1,100 nm) light to biological tissue to stimulate cellular function without thermal effects [9]. The field has progressed substantially since Karu’s foundational work in the 1980s-1990s, identifying cytochrome c oxidase (CCO) as the primary mitochondrial chromophore [10]. Hamblin and colleagues subsequently elucidated the downstream signaling cascades involving adenosine triphosphate (ATP) synthesis, nitric oxide (NO) release, reactive oxygen species (ROS) modulation, and transcription factor activation [11,12].

PBM has demonstrated efficacy across diverse pain conditions, including temporomandibular disorders [13], fibromyalgia [14], and musculoskeletal pain [15]. These conditions share features of inflammation, tissue hypoxia, and altered mitochondrial function, all of which are addressable through PBM’s established mechanisms. The expansion of PBM to primary dysmenorrhea represents a logical application given the condition’s prostaglandin-driven inflammatory pathophysiology and the uterine ischemia that characterizes painful menstruation.

Rationale: from clinical efficacy to mechanistic understanding

A recent systematic evaluation has established the clinical efficacy of PBM for primary dysmenorrhea. Ang et al. synthesized evidence from 12 randomized controlled trials involving 645 participants, demonstrating significant pain reduction with low-level light therapy (LLLT) compared to sham treatment (MD = −4.02; 95% CI = −7.21 to −0.82; p = 0.01) [16]. Similarly, LLLT demonstrated comparable efficacy to COCs with superior tolerability. However, “variations in treatment protocols contributed to heterogeneity and hindered identification of optimal parameters” [16].

This mechanistic heterogeneity reflects a fundamental gap: clinical efficacy has outpaced mechanistic understanding. While PBM is known to work for dysmenorrhea, the specific pathways mediating therapeutic benefit, and their implications for protocol optimization, remain incompletely characterized. This review addresses that gap by synthesizing biomarker evidence from clinical trials with established PBM mechanism science to construct a coherent mechanistic framework. Such a framework is essential for rational protocol development, biomarker-guided patient selection, and the design of next-generation clinical trials.

## Review

Methodology

A narrative literature review was conducted using PubMed, Scopus, and Web of Science databases in September 2025. Search terms included “photobiomodulation,” “low-level light therapy,” “primary dysmenorrhea,” “prostaglandins,” “cytochrome c oxidase,” and “metabolomics.” Randomized controlled trials, mechanistic studies, and relevant systematic reviews published in English were included. Priority was given to studies reporting biochemical or metabolomic outcomes. No formal quality scoring was performed due to the narrative design of the study.

Results

Summary of Clinical Evidence

Efficacy findings: The clinical evidence base for PBM in primary dysmenorrhea has been comprehensively synthesized by Ang et al., to which readers are referred for a detailed analysis of efficacy [16]. Briefly, a meta-analysis of three randomized controlled trials demonstrated statistically significant pain reduction with LLLT compared to sham at 12 weeks (n = 150; MD = −4.02; 95% CI = −7.21 to −0.82; p = 0.01). LLLT also demonstrated superiority over oral contraceptives at multiple timepoints (week 4: MD = 1.41; week 8: MD = 1.17; week 12: MD = 0.91; all p < 0.001).

What is noteworthy for mechanistic purposes is the 100% directional consistency across studies: all trials with biomarker or clinical endpoints demonstrated treatment effects favoring active PBM over comparators. This consistency suggests a reliable biological effect rather than a methodological artifact.

High-intensity laser therapy (HILT) at 1,064 nm demonstrated comparable efficacy to LLLT in one direct comparison [17], suggesting that multiple wavelengths may be therapeutically active, albeit potentially through different mechanisms given the distinct absorption characteristics.

Safety Profile

Across the trials included in recent synthesis, zero serious adverse events were attributed to PBM [16,18-21]. Minor adverse events (transient skin irritation from adhesive patches) occurred in <5% of participants and resolved spontaneously. This exceptional safety profile, particularly compared to NSAID-associated gastrointestinal effects and COC-associated hormonal disruption, positions PBM as an attractive alternative for patients with contraindications to or preferences against pharmacological management.

Primary Photoreceptor: Cytochrome c Oxidase

The mechanistic foundation of PBM rests on the identification of CCO (Complex IV of the mitochondrial electron transport chain) as the primary chromophore for red and near-infrared light [10,22]. CCO contains four redox-active metal centers: two copper centers (CuA and CuB) and two heme groups (heme a and heme a3). These metal centers absorb photons in the red (620-680 nm) and near-infrared (760-825 nm) spectral regions, with a peak absorption at approximately 620 nm, precisely matching the 610-630 nm wavelengths, which demonstrates optimal clinical efficacy in dysmenorrhea trials [10,11].

Upon photon absorption, CCO undergoes electronic excitation that modulates its redox state and accelerates electron transfer within the respiratory chain [11,23]. A critical consequence of this activation is the photodissociation of NO from the CCO heme centers. Under physiological or pathological conditions, NO competitively inhibits CCO by binding to the same site as oxygen, thereby reducing cellular respiration and ATP synthesis [12]. The displacement of NO by photon absorption restores oxygen binding, enhances electron transport, increases mitochondrial membrane potential, and, ultimately, elevates ATP production [11,12].

This mechanism directly addresses the uterine pathophysiology of dysmenorrhea. The intense uterine contractions and vasoconstriction induced by prostaglandins create tissue hypoxia and energy deficit. Enhanced mitochondrial function via CCO activation provides the energetic substrate for cellular repair, while released NO promotes vasodilation, potentially ameliorating uterine ischemia. The wavelength specificity of clinical efficacy (610-630 nm) thus reflects the absorption spectrum of the primary photoreceptor.

Prostaglandin Modulation: Direct Biomarker Evidence

Three clinical studies have directly measured prostaglandin levels following PBM treatment in dysmenorrhea, providing the most direct evidence for mechanistic effects [24-26]. First, Thabet et al. measured serum PGF2α using enzyme-linked immunosorbent assay in 52 women randomized to HILT (1,064 nm) versus pulsed electromagnetic field therapy [24]. The HILT group demonstrated a significant reduction in PGF2α levels (p < 0.0001) concurrent with 78.1% improvement in pain scores. This represents evidence that PBM is associated with reduced levels of the primary prostaglandin mediator of uterine hypercontractility, the fundamental driver of dysmenorrheic pain.

Second, Zhu et al. compared LLLT (630 nm) with COCs in 156 women, measuring both PGE2 and NO [25]. Both treatments significantly reduced PGE2 (LLLT: −109.57 ± 3.99 pg/mL; COC: −118.11 ± 12.93 pg/mL; p = 0.51 between groups), demonstrating that LLLT achieves prostaglandin suppression comparable to hormonal therapy through a different mechanism. Notably, LLLT maintained this effect without the systemic hormonal changes induced by COC.

Third, Wang et al. conducted metabolomic analysis using ultra-performance liquid chromatography-tandem mass spectrometry in 69 women from the same trial population, identifying 76 differential metabolites following LLLT treatment [26]. Prostaglandin D2 (PGD2) was significantly downregulated, extending the evidence of prostaglandin suppression beyond PGF2α and PGE2 to the broader prostanoid family. Table 1 reports the biomarker evidence from these three trials.

**Table 1:** Biomarker evidence from the three clinical trials included in the review. HILT = high-intensity laser therapy; LLLT = low-level light therapy; PGF2α = prostaglandin F2-alpha; PGE2 = prostaglandin E2; PGD2 = prostaglandin D2; NO = nitric oxide; COC = combined oral contraceptives

Study	Sample	Intervention	Biomarker	Change	Significance
Thabet et al. (2017) [24]	n = 52	HILT 1,064 nm	PGF2α	↓	p < 0.0001
Zhu et al. (2022) [25]	n = 156	LLLT 630 nm	PGE2	↓ 109.57 pg/mL	p < 0.001
Zhu et al. (2022) [25]	n = 156	LLLT 630 nm	NO	↑ (significant increase)	p < 0.05
Wang et al. (2023) [26]	n = 69	LLLT 630 nm	PGD2	↓	p < 0.001
Wang et al. (2023) [26]	n = 69	LLLT 630 nm	Biliverdin	↑	p < 0.001
Wang et al. (2023) [26]	n = 69	LLLT 630 nm	Cortisol	Stable (vs. ↑ with COC)	p < 0.05

The convergence of these findings (PGF2α, PGE2, and PGD2 all reduced following PBM) provides compelling evidence that PBM exerts a coordinated reduction in prostaglandin levels. Unlike NSAIDs, which directly inhibit COX enzymes, PBM appears to modulate upstream pathways, potentially through effects on arachidonic acid metabolism.

Parameter Patterns

Across the two studies in this review demonstrating efficacy, clear parameter patterns emerged [25,26]. Table 2 depicts the parameter patterns across these studies.

**Table 2:** Parameter patterns among efficacy studies.

Parameter	Most efficacious protocol
Wavelength	610–630 nm (red light)
Application site	CV4 (Guanyuan) and CV6 (Qihai) acupoints
Session duration	20 minutes
Frequency	Daily for 5 consecutive days before/during menstruation
Treatment cycles	3 menstrual cycles (12 weeks)
Energy density	3–12 J/cm²

Biliverdin Pathway: A Hypothesis-Generating Finding

Wang et al.’s metabolomic analysis identified biliverdin as significantly upregulated following LLLT treatment [26]. This finding opens an entirely novel mechanistic pathway not previously associated with PBM in dysmenorrhea.

Biliverdin is the immediate product of heme degradation by heme oxygenase-1 (HO-1). It possesses potent anti-inflammatory properties, including reduction of leukocyte infiltration, suppression of complement activation, and antioxidant activity through the biliverdin-bilirubin redox cycle [27,28]. Importantly, biliverdin has demonstrated analgesic effects in inflammatory pain models through its anti-inflammatory and antioxidant properties, suggesting direct relevance to dysmenorrhea symptom relief [28].

The upregulation of biliverdin following PBM likely reflects increased HO-1 activity, which is a known downstream effect of PBM-induced signaling cascades [29]. HO-1 is transcriptionally activated by the nuclear factor erythroid 2-related factor 2 (Nrf2) pathway, which responds to the transient ROS burst generated by enhanced mitochondrial activity following CCO activation [11,29]. The HO-1/biliverdin axis thus represents a plausible secondary anti-inflammatory pathway activated by PBM that may complement direct prostaglandin suppression.

This finding has important clinical implications. Biliverdin levels could potentially serve as a biomarker for treatment response, enabling personalized assessment of PBM efficacy. Furthermore, interventions that enhance HO-1 expression (such as certain dietary compounds) might synergize with PBM, though this hypothesis requires testing.

Lipid Metabolism Pathways: Low-Level Light Therapy-Specific Signatures

Beyond individual metabolites, pathway analysis in the Wang et al. study revealed coordinated changes in lipid metabolism [26]. Analysis of lipid metabolism revealed three key pathways significantly enriched following LLLT. First, glycerophospholipid metabolism showed the highest impact, because glycerophospholipids are major constituents of cell membranes and precursors for signaling molecules; their modulation suggests PBM effects on membrane stability and cell signaling capacity. Second, arachidonic acid metabolism was enriched, which directly connects to prostaglandin synthesis as arachidonic acid is the substrate for COX-mediated prostanoid production. Modulation of this pathway upstream of COX could explain the coordinated reduction in multiple prostaglandins (PGD2, PGE2, PGF2α) without direct COX inhibition. Finally, linoleic acid metabolism was uniquely altered only in the LLLT group, not in COC-treated patients [26]. Linoleic acid is an essential omega-6 fatty acid that serves as a precursor to arachidonic acid; its selective modulation by LLLT suggests a mechanism distinct from hormonal suppression, potentially involving effects on fatty acid elongation and desaturation enzymes.

The LLLT-specific signature in linoleic acid metabolism is mechanistically significant. It indicates that LLLT does not simply mimic the effects of hormonal therapy but rather engages a distinct pathway that may offer additive benefits when combined with other interventions. This pathway also provides a potential explanation for why LLLT demonstrates comparable efficacy to COC without inducing the hormonal changes (increased cortisol, testosterone glucuronide) observed with COC [26]. Table 3 depicts the metabolic pathways modulated by LLLT as reported by Wang and colleagues [26].

**Table 3:** Metabolic pathways modulated by LLLT from Wang et al. [26]. LLLT = low-level light therapy

Pathway	Impact score	LLLT-specific?	Relevance to dysmenorrhea
Glycerophospholipid metabolism	Highest	No	Cell membrane stability, signaling
Linoleic acid metabolism	High	Yes	Arachidonic acid precursor
Arachidonic acid metabolism	High	No	Direct prostaglandin precursor
Steroid hormone biosynthesis	Moderate	No	Hormonal regulation

Discussion

Integration: The Mechanistic Cascade

Synthesizing the evidence, the following is proposed mechanistic cascade for PBM in primary dysmenorrhea. The primary event involves photon absorption by CCO (peak absorption ~620 nm). Immediate consequences include photodissociation of NO from CCO (restoring cellular respiration), enhanced electron transport (elevating ATP synthesis), and a transient ROS increase (activating signaling pathways).

**Figure 1:**
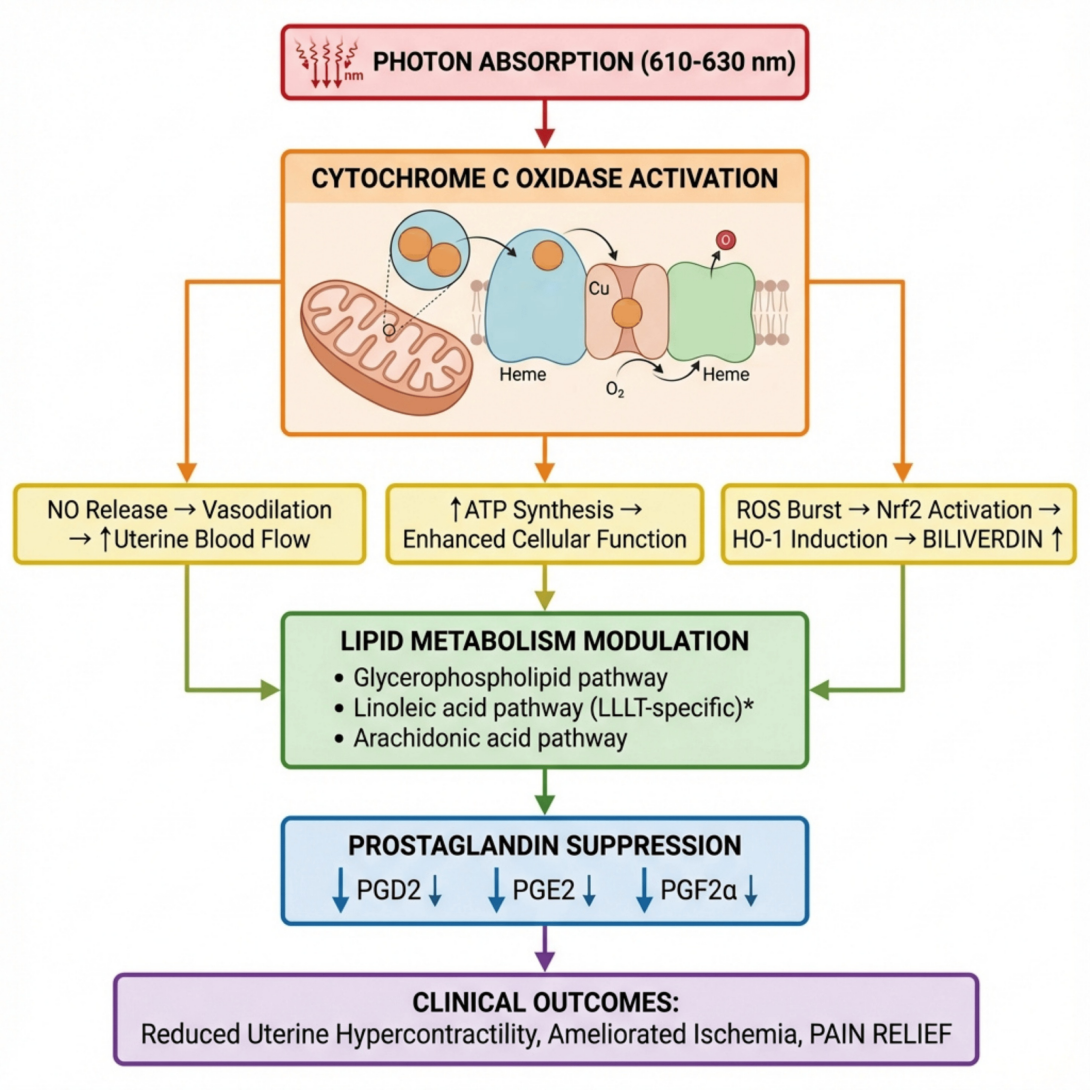
Proposed mechanistic cascade underlying photobiomodulation in primary dysmenorrhea. Proposed mechanistic cascade for photobiomodulation in primary dysmenorrhea. Photons at 610–630 nm are absorbed by cytochrome c oxidase (CCO) in the mitochondrial respiratory chain. This primary event triggers nitric oxide (NO) release (promoting vasodilation), enhanced ATP synthesis (supporting cellular function), and a transient reactive oxygen species (ROS) increase that activates Nrf2/HO-1 signaling (leading to biliverdin production). Secondary effects include modulation of lipid metabolism pathways (glycerophospholipid, linoleic acid, arachidonic acid), ultimately resulting in reduced circulating prostaglandins (PGF2α, PGE2, PGD2) and biliverdin-mediated anti-inflammatory effects. Clinical outcomes include reduced uterine hypercontractility, ameliorated ischemia, and pain relief. Conceptual framework created by the authors based on evidence from references [10,11,22-26,29].

Secondary signaling involves NO release promoting vasodilation and improved uterine blood flow; ROS activating Nrf2 and HO-1 induction leading to biliverdin production; and membrane lipid changes altering fatty acid metabolism. Observed downstream effects include reduced circulating prostaglandins (PGD2, PGE2, PGF2α), hypothesized biliverdin-mediated anti-inflammatory effects, and modulation of lipid metabolism pathways. Clinical outcomes include reduced uterine hypercontractility, ameliorated uterine ischemia, decreased pain receptor sensitization, and pain relief. Figure 1 illustrates the proposed mechanistic cascade for PBM in PD. 

Comparison With Other Pain Conditions

The mechanistic pathways identified in dysmenorrhea research parallel findings in other pain conditions, supporting a shared biological framework for PBM analgesia.

In temporomandibular disorders, PBM reduces inflammatory cytokines (interleukin-1β, tumor necrosis factor-alpha) and prostaglandins while enhancing tissue repair through ATP-mediated cellular function [13,30]. The anti-inflammatory profile mirrors the prostaglandin suppression observed in dysmenorrhea trials.

In fibromyalgia, whole-body PBM produces systemic regulation of nociceptive pathways and autonomic function, with improvements in pain, fatigue, and quality of life [14]. Wang et al.’s finding of cortisol stability following LLLT (vs. elevation with COC) suggests PBM may avoid hypothalamic-pituitary-adrenal axis stress responses, potentially relevant to the central sensitization component of chronic pain conditions [26].

In musculoskeletal pain, PBM accelerates tissue repair through enhanced collagen synthesis and reduced oxidative stress, effects mediated through the same CCO/ATP pathway [15,31]. The convergence across conditions supports CCO activation as a universal mechanism, with downstream effects varying according to tissue-specific pathophysiology.

Implications for Protocol Optimization

Wavelength selection: The mechanistic framework provides a clear rationale for wavelength selection. CCO exhibits absorption peaks at approximately 620 nm (red) and 820 nm (near-infrared), corresponding to its copper and heme centers [10,11]. The clinical evidence favoring 610-630 nm aligns precisely with the red absorption peak.

The efficacy of HILT at 1,064 nm [17,24] is less readily explained by CCO absorption. At this wavelength, water absorption increases and CCO absorption is minimal. The mechanism underlying 1,064 nm therapeutic effects remains currently uncertain; there is currently not sufficient evidence to favor any specific explanation. Possibilities include different chromophore targets (such as water or lipids), deeper tissue penetration compensating for reduced absorption, or photothermal effects despite nominal classification as “non-thermal.” Until comparative mechanistic studies are conducted, the biophysical basis for 1,064 nm efficacy should be considered unknown.

Consequently, for LLLT protocols, wavelengths of 610-630 nm should be prioritized based on CCO absorption characteristics and clinical evidence. Protocols deviating significantly from this range should provide a mechanistic justification.

Application site selection: The predominant application sites in efficacious trials are acupuncture points CV4 (Guanyuan) and CV6 (Qihai), located on the lower abdominal midline [18-21,25,26]. This choice reflects the integration of traditional Chinese medicine concepts with modern PBM science.

From a mechanistic perspective, these sites offer advantages. First, regarding anatomical proximity, CV4 and CV6 overlie the uterus without intervening bone, minimizing optical scattering and maximizing photon delivery to target tissue. Second, regarding penetration depth, at 630 nm, tissue penetration depth is approximately 1-3 mm for effective therapeutic dosing [32]. The thin abdominal wall in this region may allow regional neurovascular and indirect uterine effects. Third, regarding innervation considerations, the hypogastric plexus innervating the uterus courses through this region. PBM effects on nerve function (through NO release and reduced inflammation) may modulate pain signaling independent of direct uterine tissue effects.

Based on this evidence, the CV4/CV6 application should be considered the default approach. Direct lower abdominal application over the uterine fundus represents a reasonable alternative that may improve accessibility for self-treatment.

Dosimetry and treatment timing: The energy densities used across efficacious trials (3-12 J/cm²) fall within the range associated with stimulatory effects per the Arndt-Schulz law (biphasic dose response) [11]. Below-threshold dosing fails to activate cellular responses, while excessive dosing may produce inhibitory effects through oxidative stress.

Treatment timing (five days before and/or during menstruation) aligns with the pathophysiology. Prostaglandin production increases in the late luteal phase as progesterone withdrawal triggers endometrial breakdown [2]. Initiating PBM before symptom onset may prevent the prostaglandin surge, while treatment during menstruation provides acute intervention.

Therefore, the best-supported protocol of 3 J/cm² for 20-minute sessions, applied daily for five days before/during menstruation across three menstrual cycles, should serve as the reference standard for future trials seeking to optimize specific parameters.

Skin Phototype Considerations

A critical evidence gap concerns the influence of skin melanin on PBM efficacy. Melanin absorbs red light, potentially reducing photon delivery to deeper tissues in individuals with higher skin melanin content [33]. All existing dysmenorrhea trials were conducted in East Asian populations (Fitzpatrick skin types II-IV), with no studies from populations with very light (type I) or very dark (types V-VI) skin.

Computational modeling suggests that at 630 nm, melanin absorption may reduce effective tissue dose in darker skin types, though the magnitude of this effect requires empirical validation in clinical populations [33]. This could necessitate increased surface power density or longer treatment durations to achieve equivalent tissue effects in diverse populations.

To address this gap, future trials might consider stratifying by Fitzpatrick skin type and consider adjusting dosimetry accordingly. This may be important for the global generalizability of PBM protocols.

Research Priorities

Mechanistic studies: First, no dysmenorrhea trials have systematically varied wavelength, power density, or duration to establish dose-response curves. Such studies are essential for optimizing protocols and would provide direct evidence for or against the proposed mechanistic framework. Second, Wang et al.’s metabolomic findings require replication with larger samples and pre-specified hypotheses. Biliverdin, in particular, warrants investigation as a potential response biomarker that could enable personalized treatment assessment. Finally, the relative contributions of immediate (vasodilation) versus delayed (gene expression changes) effects remain unclear. Time-course studies measuring biomarkers at multiple points during and after treatment would clarify which mechanisms predominate.

Clinical trials: A direct head-to-head comparison of 630 nm LLLT versus 1,064 nm HILT with biomarker endpoints would clarify whether these modalities work through shared or distinct mechanisms. Trials in diverse European, African, and American populations are urgently needed to establish global generalizability and inform potential dosimetric adjustments for varying skin phototypes. Given LLLT’s distinct mechanism (vs. COC), combination therapy trials could assess whether concurrent use provides additive benefits beyond either intervention alone.

Implementation research: While portable LED devices enable home-based treatment, adherence, proper application technique, and real-world effectiveness require pragmatic trial evaluation. Furthermore, device costs represent an upfront investment potentially offset by reduced pharmaceutical use. Formal cost-effectiveness analysis would inform healthcare policy decisions.

## Conclusions

This narrative review integrates clinical biomarker, metabolomic, and mechanistic evidence to support a biologically coherent model through which PBM may alleviate PD. Across available studies, PBM, particularly LLLT at 610-630 nm, was consistently associated with reductions in multiple prostaglandins and modulation of lipid metabolic pathways directly implicated in prostaglandin synthesis, findings that align with established mitochondrial signaling mechanisms involving CCO and provide a rationale for observed wavelength specificity. The convergence of prostaglandin suppression, lipid pathway modulation, and mitochondrial activation distinguishes PBM from pharmacologic and hormonal therapies, suggesting a mechanistically distinct non-pharmacologic approach to dysmenorrhea management. Future research should prioritize dose-response optimization, replication of metabolomic findings, and validation of candidate biomarkers to refine treatment protocols and improve generalizability across populations.
